# Illness acceptance and adaptation in adults with an intestinal stoma in Poland: a cross-sectional pilot study

**DOI:** 10.3389/fpubh.2026.1757185

**Published:** 2026-05-29

**Authors:** Agnieszka Pluta, Patrycja Szukalska, Mariola Głowacka, Marzena Humańska

**Affiliations:** 1 Faculty of Health Sciences, Ludwik Rydygier Collegium Medicum in Bydgoszcz of the Nicolaus Copernicus University in Toruń, Bydgoszcz, Poland; 2 Multispecialist Hospital named after Dr. Ludwik Błażek, Inowrocław, Poland; 3 Collegium Medicum, The Mazovian University, Płock, Poland

**Keywords:** adaptation, illness acceptance, nursing care, psychosocial support, quality of life, stoma

## Abstract

**Aim:**

To examine the level of illness acceptance among adults living with an intestinal stoma and to identify factors associated with adaptation.

**Design:**

Quantitative, cross-sectional, pilot study.

**Methods:**

A cross-sectional study was conducted in 2023 at the Multispecialist Hospital in Inowrocław, Poland, among 60 adult patients with a stoma. A self-designed questionnaire, the Barthel Index, and the Acceptance of Illness Scale (AIS) were applied. Group comparisons were conducted using the Mann–Whitney U test, Kruskal–Wallis test, and *χ*^2^ test; a significance level of *p* < 0.05 was adopted. This study is reported by the STROBE checklist for cross-sectional studies.

**Results:**

The mean AIS score was 23.86 ± 7.07 points, indicating moderate acceptance overall. Low, moderate, high, and very high acceptance levels were observed in 31.7%, 56.7%, 11.6%, and 11.6% of participants, respectively. The highest AIS scores were reported by patients aged 41–55 years, while the lowest were in those aged ≥71 years. Age was significantly associated with illness acceptance, but the type of stoma was not. Family support was reported by 71.7% of participants, and 55% evaluated the impact of hospital education positively.

**Conclusion:**

Age, education, and social support are critical determinants of adaptation to life with a stoma. Nursing practice should address not only technical aspects of stoma care but also psychosocial and relational dimensions. This study highlights that hospital-based education plays a key role in improving psychosocial adaptation and illness acceptance, which may translate into better quality of life and reduced care burden. Strengthening stoma care education is particularly important in an aging population, where early support may help prevent complications, rehospitalizations, and long-term functional decline.

## Highlights

- This study addresses the problem of insufficient adaptation and illness acceptance among stoma patients.
- Findings highlight the importance of nursing, education, and family support in improving quality of life.
- Results can inform clinical practice by emphasizing the value of holistic nursing care.

## 1 Introduction

The creation of an intestinal stoma significantly impacts a patient’s physical, psychological, and social well-being. The literature highlights that adapting to life with a stoma involves a complex psychological process in which illness acceptance is crucial (1–3). Illness acceptance is understood as how well a patient adjusts to the limitations and changes caused by a chronic condition, including shifts in body image and social roles (4).

Studies indicate that stoma patients frequently experience a range of negative emotions, such as shame, guilt, anxiety, and depression, which may hinder their everyday functioning (1, 5). Commonly reported challenges include reduced self-esteem, limitations in occupational and sexual life, and difficulties in maintaining social relationships (1, 3, 6). Social support is recognized as a crucial protective factor against adverse psychological outcomes. Equally important is health education, which prepares patients for self-care and provides psychological support both in the perioperative period and throughout the further course of treatment (1, 3).

Sociodemographic factors such as age, sex, marital status, socioeconomic position, and educational level have also been shown to significantly influence illness acceptance. Younger patients, those living alone, and individuals with lower socioeconomic status often report lower acceptance and greater difficulties in adaptation (1–3). In addition, the type of stoma (colostomy or ileostomy) and the duration of living with it are factors that differentiate the degree of psychological adjustment (7, 8). Considering these variables in research enables a better understanding of adaptive mechanisms and helps identify patients who require targeted therapeutic and educational support. Findings from such studies may provide the basis for developing effective psychological support programs and interventions aimed at improving quality of life in this population.

The present study aimed to determine the level of illness acceptance (AIS) among adult patients with an intestinal stoma and to identify sociodemographic and clinical factors associated with this level.

## 2 Methods

The study was conducted by the principles of the Declaration of Helsinki. Ethical approval was obtained from the Bioethics Committee of Nicolaus Copernicus University in Toruń (No. 18/2023, dated 17 January 2023). All participants provided informed consent to take part in the study.

The research was carried out between January and July 2023 at a multispecialist hospital in Poland. The study group consisted of 60 patients with an intestinal stoma, aged 23–85 years, including 37 men and 23 women.

### 2.1 Inclusion criteria

- Age ≥ 18 years;
- presence of an intestinal stoma (colostomy or ileostomy; temporary or permanent), regardless of whether it was formed electively or emergently;
- provision of informed consent to participate in the study;
- hospitalization in the study center during the data collection period;
- ability to understand the questions and complete the questionnaires independently.

### 2.2 Exclusion criteria

- Lack of consent to participate or withdrawal of consent at any stage;
- cognitive impairment or severe mental disorders preventing informed consent or reliable questionnaire completion;
- significant sensory or communication deficits (vision, hearing, or speech) that prevented questionnaire completion despite available support.

This study was pilot and exploratory in nature, and sample size was determined by the number of patients meeting the inclusion criteria hospitalized at the center during the data collection period.

A self-designed questionnaire comprising 17 items (15 closed-ended and 2 open-ended questions) was applied. In addition, the Barthel Index was used to assess independence, and the Acceptance of Illness Scale (AIS) was employed. The AIS consists of 8 statements describing the consequences of living with a chronic condition. Responses are given on a five-point Likert scale, ranging from 1 (“strongly agree”) to 5 (“strongly disagree”). Since all items are phrased negatively, lower scores reflect poorer adaptation, whereas higher scores indicate greater illness acceptance. The total score ranges from 8 to 40 points; the higher the score, the greater the acceptance of illness and the lower the intensity of negative emotions. In this study, the following cut-off points were applied: <20 points – low level of acceptance, 20–30 points – moderate level, and >30 points – high level of acceptance. For broader context, the results were also compared with values reported in other clinical populations (9). The Polish adaptation of the AIS has demonstrated good internal consistency in validation studies (Cronbach’s alpha ≈ 0.82) (9).

The reporting of this study follows the STROBE (Strengthening the Reporting of Observational Studies in Epidemiology) guidelines for cross-sectional studies.

### 2.3 Statistical analysis

Data analysis was performed using IBM SPSS and Microsoft Excel. Before selecting statistical tests, the distribution of measurable variables was assessed using the Shapiro–Wilk test. Due to the lack of normality of the distribution, nonparametric tests were used.

Nonparametric tests were applied:

- The Mann–Whitney U test for comparisons between two groups,
- The Kruskal–Wallis test for comparisons among three or more groups.

The level of statistical significance was set at *p* < 0.05. Results are presented as means, standard deviations, and medians.

## 3 Results

The study group consisted of 60 patients with an intestinal stoma, including 37 men (61.7%) and 23 women (38.3%). The mean age of participants was 62 years. The largest subgroup comprised patients aged 56–70 years (43.3%), while the smallest subgroup included individuals younger than 40 years (6.7%). The predominant type of stoma was colostomy (81.7%), while 18.3% of patients had an ileostomy. In most cases, the stoma was formed electively (63.3%), whereas 36.7% of patients underwent emergency stoma formation (Table 1).

**Table 1:** Characteristics of the study group (*n =* 60).

Variable	*n*	%
Sex		
Male	37	61.7
Female	23	38.3
Age		
< 40 years	4	6.7
41–55 years	12	20.0
56–70 years	26	43.3
≥ 71 years	18	30.0
Education		
Primary	14	23.3
Vocational	21	35.0
Secondary	18	30.0
Higher	7	11.7
Place of residence		
Urban	37	61.7
Rural	23	38.3
Marital status		
Single	6	10.0
Married	33	55.0
Widowed	15	25.0
Divorced	6	10.0
Occupational status		
Employed (full-time)	16	26.7
Self-employed	4	6.7
Unemployed	2	3.3
Retired	32	53.3
On disability pension	6	10.0
Type of stoma		
Colostomy	49	81.7
Ileostomy	11	18.3
Mode of stoma formation		
Elective	38	63.3
Emergency	22	36.7

In terms of emotions experienced after stoma formation, sadness was the most frequently reported (43.3%), followed by anxiety (30%). Only 10% of patients reported acceptance of the situation, while 5% admitted having suicidal thoughts.

**Figure 1:**
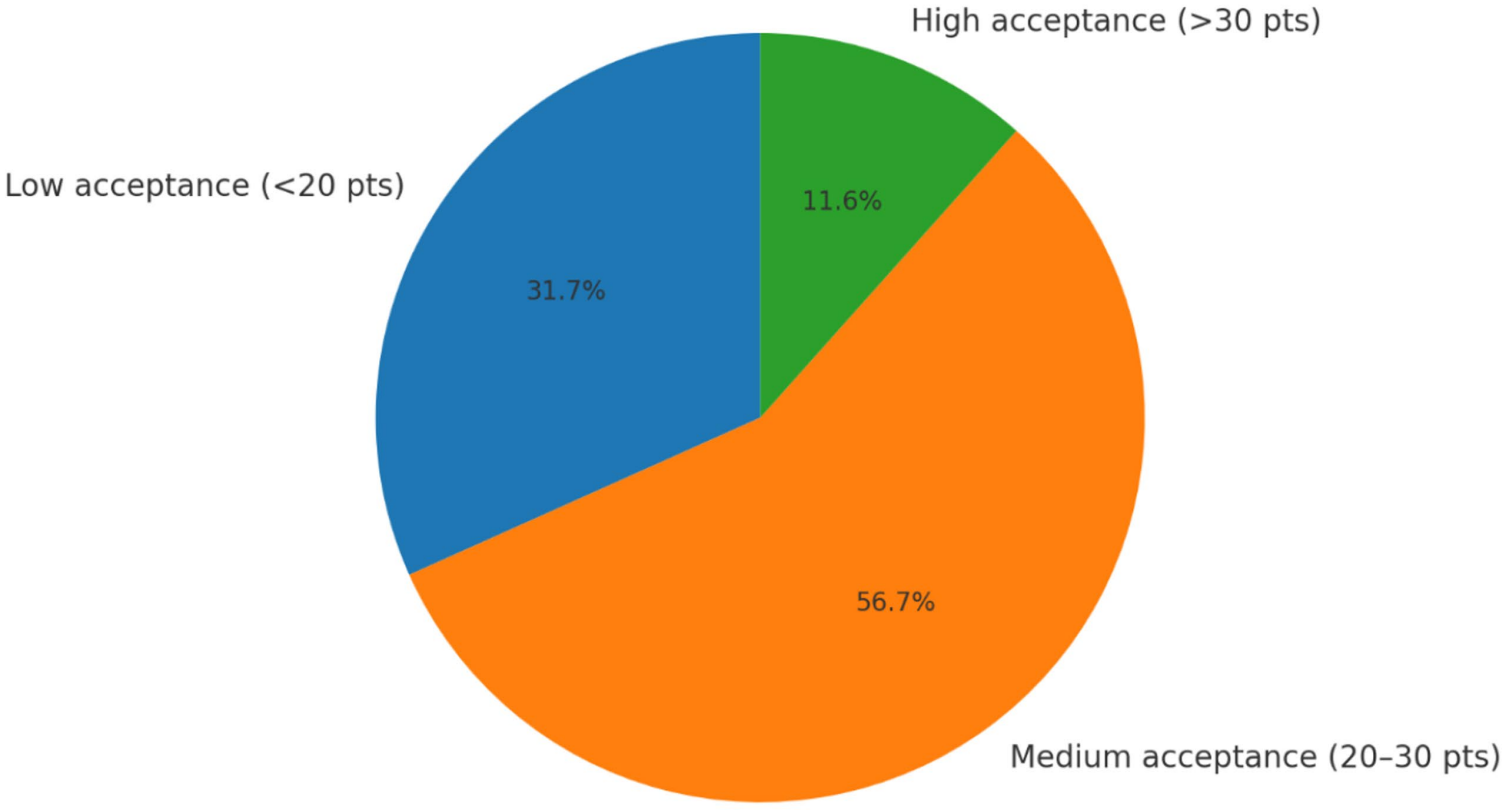
Distribution of illness acceptance levels among the study participants (*n =* 60).

Regarding the level of illness acceptance, assessed with the Acceptance of Illness Scale (AIS), the mean score was 23.86 ± 7.07 points. The minimum score was 11 and the maximum was 38. A low level of acceptance (<20 points) was found in 31.7% of patients, a moderate level (20–30 points) in 56.7%, and a high level (>30 points) in 11.6% (Figure 1).

Age was significantly associated with AIS scores (*p* = 0.042). The highest level of illness acceptance (high AIS) was observed in the 41–55 age group (33% of this group), while a moderate level predominated among patients aged 56–70 years (58%). The lowest acceptance was most frequently reported among participants over 71 years of age (33% of this group).

**Figure 2:**
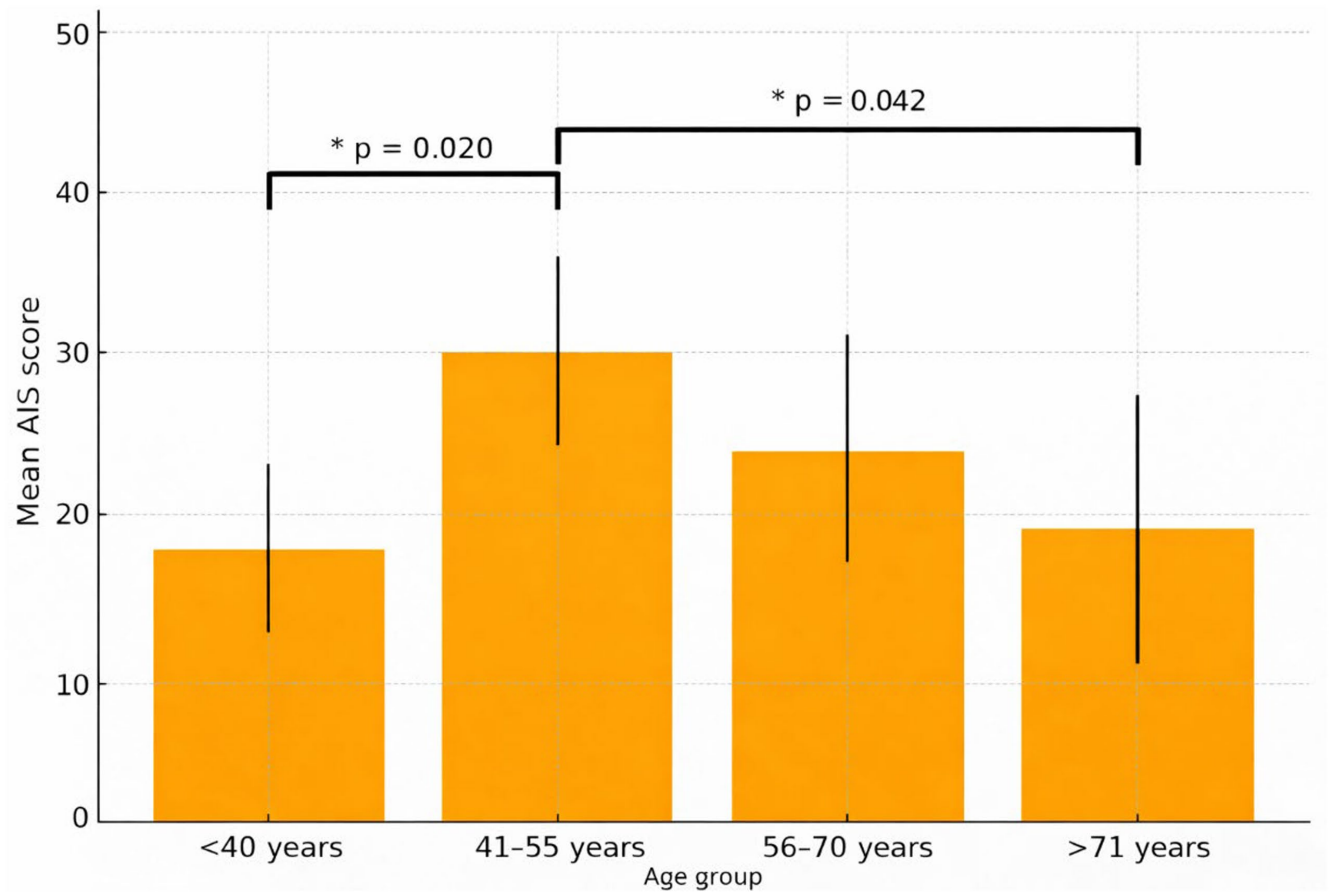
Illness acceptance (AIS) across age groups among patients with an intestinal stoma (*n =* 60).

Analysis by sex revealed that women were more likely than men to report a low level of illness acceptance (39.1% vs. 27%), whereas a high level of acceptance was more frequently observed among men (16.2%) than women (4.4%). However, chi-square analysis did not show statistically significant differences between sexes (*χ*^2^ = 2.37; *p* = 0.306) (Figure 2).

In terms of independence assessed using the Barthel Index, the mean score was 57.2 ± 27.2 points, indicating a moderate level of independence in performing daily activities. Full independence was reported by 48.3% of patients, 30% required partial assistance, and 21.7% were completely dependent on others.

Most patients (71.7%) reported receiving support from their families, 23.3% were undecided, and 5% declared a lack of such support. Regarding the impact of hospital education, 55% of respondents stated that it improved their illness acceptance, 38.3% noticed no changes, and 6.7% reported that acceptance worsened despite education.

Among the analyzed sociodemographic and clinical variables, only age was significantly associated with illness acceptance (*p* = 0.042). No statistically significant associations were observed for sex, education, type of stoma, mode of stoma formation, family support, or functional independence.

## 4 Discussion

The present study provides an assessment of illness acceptance among patients with an intestinal stoma and an analysis of factors influencing their adaptation. This discussion highlights the significance of the findings about the current state of knowledge and outlines practical implications for patient care.

Our results indicate that a moderate level of illness acceptance (mean AIS score: 23.9 ± 7.1 points) predominates among stoma patients. This finding is consistent with data reported by Ayaz-Alkaya (1), who emphasized that illness acceptance plays a crucial role in psychological adjustment, including reduced body image distress and improved mental well-being. Similarly, other researchers have observed that a higher level of illness acceptance correlates with better psychosocial functioning and quality of life in people with a stoma (2, 3, 8).

One of the key factors influencing illness acceptance is the quality and scope of health education provided during the perioperative period. In our study, 55% of patients indicated that hospital education was an important factor supporting adaptation. For example, Iovino et al. (10) described the effectiveness of a telehealth-versus-in-person self-care education programme in ostomy patients, which improved self-care, adjustment and quality of life. Similarly, Paszyńska (11) emphasised the value of perioperative education including psychosexual counselling in reducing complications, enhancing independence and improving daily functioning.

It is important to note that education should not be limited to technical care instructions but also address emotional, social, and sexual aspects (1, 11). Recent evidence demonstrates that educational programs incorporating telemedicine are particularly effective, as they provide continuous access to the healthcare team, increase patients’ sense of security, and reduce anxiety related to daily functioning (10). Some authors also highlight the value of involving multiple specialists—such as the surgeon, stoma nurse, psychologist, or sexologist—in supporting patients, as multidisciplinary input can help tailor educational and psychosocial interventions to individual needs (1, 11). Such an approach may contribute to better stoma acceptance and help reduce long-term psychological consequences, including stigma, depression, and social withdrawal (1, 5).

In our study, no significant differences in AIS were observed between patients with colostomy and ileostomy (*p* = 0.77). However, recent evidence suggests that patients with ileostomy may face a higher risk of complications, greater care-related difficulties, and heavier psychological burden compared with colostomy (12). Furthermore, longitudinal observational work demonstrates that, regardless of stoma type, patients tend to adapt psychosocially over time, with many reporting improved adjustment in the months following surgery (13, 14).

The findings of the present study confirm previous reports showing that illness acceptance among stoma patients is generally at a moderate level and is influenced by sociodemographic and clinical factors (1–3, 7, 8). However, our analysis specifically demonstrated that age significantly affects acceptance levels, which provides an important addition to existing evidence in this area. Another novel contribution is the identification of hospital education as a relevant determinant of adaptation, with more than half of the patients indicating its positive impact. Although recent publications discuss the role of education and digital support in stoma care (10, 15), our study extends these observations within the Polish context.

The originality of this study lies in highlighting the interdisciplinary role of education and family support as elements that may substantially enhance the psychosocial adaptation of stoma patients. These findings may serve as a basis for designing targeted educational programs and psychological interventions in clinical practice.

Beyond the statistical associations observed, our results also draw attention to broader dimensions of adaptation that have been described in the literature. Illness acceptance is understood not only as an individual psychological trait but as a dynamic process shaped by personal resilience, social support, and the healthcare environment (1, 5). The lower acceptance observed among older patients in our study may reflect age-related disparities in access to psychosocial and educational resources, an issue previously noted in gerontological nursing research.

From a holistic perspective, nursing care includes not only technical and clinical interventions, but also advocacy and actions that support patients’ dignity, autonomy, and social participation - dimensions emphasized in contemporary models of person-centred and relationship-based nursing. Previous studies highlight that the experience of living with a stoma often involves redefining body image, identity, and roles in daily life, and that supportive relationships with healthcare professionals and family members can play a key role in this process (1, 6).

Our findings suggest that structured education and active family involvement may facilitate not only the acquisition of self-care skills but also enhance emotional well-being and a sense of connection. By situating the results within this broader conceptual framework, illness acceptance emerges as both a clinical indicator and a psychosocial process that benefits from integrated, patient-centred approaches. Future research should therefore explore how individualized education, psychosocial support, and collaborative care models can further strengthen adaptation and quality of life in people living with an intestinal stoma.

From a public health perspective, strengthening hospital-based educational interventions may improve long-term adaptation and reduce the risk of preventable complications, rehospitalizations, and care-related difficulties. As the majority of stoma patients are older adults, integrating structured educational support into routine care pathways is essential for promoting independence, reducing system-level costs, and improving population health outcomes.

This study has several limitations. First, it was conducted in a single center, which may limit the generalizability of the findings to other clinical settings or populations. Second, due to its cross-sectional design, causal relationships between the analyzed variables and illness acceptance cannot be established.

Potential sources of bias should also be considered. Data were collected using self-reported questionnaires, which may be subject to response bias and social desirability bias. The questionnaires were administered during hospitalization, which could have influenced participants’ responses due to their clinical condition or perceived dependence on healthcare staff.

To minimize bias, participants completed the questionnaires independently, and when assistance was required, it was limited to reading questions and recording answers without interpretation or suggestion. The researchers aimed to maintain a neutral and non-judgmental approach during all interactions with participants.

Additionally, selection bias cannot be excluded, as the study included only patients hospitalized in a single center who met the inclusion criteria and agreed to participate.

Future multicenter longitudinal studies with larger samples are needed to confirm these findings.

## 5 Conclusion

1. The level of illness acceptance was moderate; acceptance was significantly associated with age (lower among the oldest patients), while sex differences represented nonsignificant tendencies. Descriptive analysis indicated that higher AIS scores co-occurred with positive evaluation of hospital education and reported family support (without statistical significance).
2. Hospital education focused on self-care and psychosocial support should be an integral part of care for stoma patients.
3. In care planning, family support resources—reported by the majority of patients—should be actively utilized.
  These findings underscore the public health importance of structured hospital education and family engagement, which can enhance patients’ adaptation, reduce care burden, and support healthy aging in the growing population of individuals living with an intestinal stoma.

### 5.1 Recommendations

1. It is recommended to implement structured educational programs for stoma patients, covering not only technical aspects but also psychological and social dimensions, including preparation for changes in daily and sexual life.
2. In clinical practice, special attention should be paid to groups at higher risk of lower illness acceptance—particularly women and older patients—by offering individual psychological consultations and support from stoma care nurses.
3. Future research should address the long-term effects of education and the impact of psychological support on adaptation, as well as explore aspects that have so far been overlooked, such as sleep quality, sexual life, and the sense of stigmatization.

### 5.2 Study limitations

This study was single-center and cross-sectional in nature. Future research should involve multicenter studies with larger samples and multivariate analyses.

## Data Availability

The raw data supporting the conclusions of this article will be made available by the authors, without undue reservation.
